# Meiotic Recombination Analyses in Pigs Carrying Different Balanced Structural Chromosomal Rearrangements

**DOI:** 10.1371/journal.pone.0154635

**Published:** 2016-04-28

**Authors:** Nicolas Mary, Harmonie Barasc, Stéphane Ferchaud, Aurélia Priet, Anne Calgaro, Anne-Marie Loustau-Dudez, Nathalie Bonnet, Martine Yerle, Alain Ducos, Alain Pinton

**Affiliations:** 1 INRA, UMR1388 Génétique, Physiologie et Systèmes d’Elevage, Castanet-Tolosan, France; 2 Université de Toulouse INPT ENSAT, UMR1388 Génétique, Physiologie et Systèmes d’Elevage, Castanet-Tolosan, France; 3 Université de Toulouse INPT ENVT, UMR1388 Génétique, Physiologie et Systèmes d’Elevage, Toulouse, France; 4 UE1372 GenESI Génétique, Expérimentation et Système Innovants, Surgères, France; University of California, San Francisco, UNITED STATES

## Abstract

Correct pairing, synapsis and recombination between homologous chromosomes are essential for normal meiosis. All these events are strongly regulated, and our knowledge of the mechanisms involved in this regulation is increasing rapidly. Chromosomal rearrangements are known to disturb these processes. In the present paper, synapsis and recombination (number and distribution of MLH1 foci) were studied in three boars (*Sus scrofa domestica*) carrying different chromosomal rearrangements. One (T34he) was heterozygote for the t(3;4)(p1.3;q1.5) reciprocal translocation, one (T34ho) was homozygote for that translocation, while the third (T34Inv) was heterozygote for both the translocation and a pericentric inversion inv(4)(p1.4;q2.3). All three boars were normal for synapsis and sperm production. This particular situation allowed us to rigorously study the impact of rearrangements on recombination. Overall, the rearrangements induced only minor modifications of the number of MLH1 foci (per spermatocyte or per chromosome) and of the length of synaptonemal complexes for chromosomes 3 and 4. The distribution of MLH1 foci in T34he was comparable to that of the controls. Conversely, the distributions of MLH1 foci on chromosome 4 were strongly modified in boar T34Inv (lack of crossover in the heterosynaptic region of the quadrivalent, and crossover displaced to the chromosome extremities), and also in boar T34ho (two recombination peaks on the q-arms compared with one of higher magnitude in the controls). Analyses of boars T34he and T34Inv showed that the interference was propagated through the breakpoints. A different result was obtained for boar T34ho, in which the breakpoints (transition between SSC3 and SSC4 chromatin on the bivalents) seemed to alter the transmission of the interference signal. Our results suggest that the number of crossovers and crossover interference could be regulated by partially different mechanisms.

## Introduction

Meiosis is an essential phase in the life cycle of sexually reproducing eukaryotes. At the chromosome level, several steps are required for the normal course of meiosis: pairing of the chromosomes, which become physically close to one another, synapsis, which leads to the formation of a proteinaceous structure known as the synaptonemal complex (SC), closely associating homologous chromosomes, and the segregation of one chromosome from each pair in the two daughter cells [1]. All these steps are intimately linked to the tightly regulated meiotic recombination process.

Recombination is initiated by programmed double-strand breaks (DSB). Several models for DSB repair have been proposed, leading to crossover (CO) and non-crossover (NCO) events at the end of pachytene [2]. NCO are thought to outnumber CO by a factor of ten in mice and humans [1], but what orientates the repair mechanisms towards CO or NCO events, and at what stage, remains elusive. Various regulatory mechanisms, operating at different levels, have evolved to ensure a correct number of CO per cell. Homeostasis allows a minimum number of CO to be maintained when the number of DSB is reduced [3]. Other mechanisms, source of the "obligatory CO" rule, ensure that at least one CO per chromosome is formed, independently of its size [4]. Finally, a long known phenomenon called "crossover interference" affects the distances between CO along the different chromosomes by discouraging CO formation in the vicinity of others [5]. Interference assumes the existence of some form of communication along the chromosomes. The mechanisms involved in that communication are not well understood, but some authors have reported that interference can act over long distances, and is propagated through the centromeres [6]. The location of CO events is also well regulated. For instance, some regions are more prone to CO (distal ends of acrocentric or metacentric chromosomes) than others (pericentromeric regions of metacentric chromosomes) [7,8]. Moreover, short genomic regions of about 1–2 kb, called "recombination hotspots", are mostly involved in recombination events [9]. In some species, including mouse and Man, a specific, rapidly evolving gene (Prdm9), has been shown to have a major role in specifying recombination hotspots [1].

As illustrated in the previous paragraphs, and thanks to huge research efforts, our knowledge of the (molecular) biology of meiosis has greatly improved over the years. Nevertheless, as stated by Mercier *et al*. (2015) [10], different points, such as the origins of the recombination landscape (regulation of the CO rates), the function of the SC, or the mechanisms of CO interference, remain elusive.

Structural chromosomal rearrangements, like chromosome fusions, translocations or inversions, can occur naturally or be created experimentally. They can be responsible for original pairing configurations, and/or synaptic defects. They can also modify the length, the structure, or the DNA content of whole chromosomes (or chromosome arms/regions), as well as the configuration of the chromatin. Therefore, they can be used as tools to provide valuable information on different aspects of meiosis regulation. Hillers and Villeneuve (2003) [11], for instance, used chromosome fusions in *C*. *elegans* to demonstrate that the meiotic chromosome axes, or SC, might represent relevant functional units for CO regulation (the presence of an axial discontinuity on only one partner chromosome in the heterozygotes can partially, but not completely, disrupt the ability to communicate the presence of a (nascent) CO and/or to discourage others in response). This idea was later confirmed by Libuda *et al*. (2013) [12] who demonstrated that a partial (RNAi-based) depletion of SC proteins attenuated CO interference, thereby increasing CO rates and reducing the effective distance over which interference operates. Still in *C*. *elegans*, McKim *et al*. (1988) [13] and Alpi *et al*. (2003) [14], among others, demonstrated that the suppression of recombination in translocation heterozygotes is severe and extensive. Crossing over does not occur in the translocation regions, which is why they are widely used as genetic balancers. As hypothesized by McKim *et al*. (1988) [13] this recombination suppression would be the result of homologous sequences failing to pair.

Studies involving chromosomal rearrangements have also been carried out in organisms exhibiting the canonical meiotic program, for instance in plants, where the ability of a given chromosome region to form chiasmata has been studied using structural chromosome mutants in wheat, rye and *Arabidopsis* (see Naranjo (2015) [15] for a review). Ederveen *et al*. (2015) [16], for instance, demonstrated in *Arabidopsis* that changes to the structural integrity of chromosomes (deletions, inversions) drastically alter the recombination landscape: recombination was silenced over the length of the structural change, while recombination over the whole chromosomes remained unchanged (strictly intrachromosomal compensation effect, also described in *C*. *elegans* by McKim *et al*. (1988) [13], and Zetka and Rose (1992) [17]). Overall, some results in plants suggest that the recombigenic capacity of a given segment is mainly determined by its position along the telomere-centromere axis, while other results have shown that chiasma formation and distribution is not conditioned by the position but depends mainly on the DNA sequence, or chromatin organization. For example, CO are mainly confined to gene rich regions in wheat or barley, while the high degree of heterochromatin packaging might be a factor limiting the accessibility to such chromatin regions of proteins either involved in the formation of DSBs, or in the subsequent recombinational repairing process, thereby inhibiting CO formation [15].

In Humans, the chiasma frequency distribution has been studied in a relatively large number of male carriers (reviewed by Hulten (2011) [18]; most studies dating back to the 70’s and 80’s). The most striking deviation from the situation in human males with normal karyotypes is a significant increase in the frequency of chiasmata localized within the interstitial segment, i.e., the chromosome segment positioned in between the breakpoint and the centromere. In stark contrast to the normal situation in non-acrocentric chromosomes, there is a tendency for chiasmata to occupy positions near to/adjacent to the centromere, and for the CO interference distance over the centromere to be substantially reduced.

Chromosomal rearrangements have also been considered in numerous meiotic studies involving rodents species (see for instance the recent papers by Dumas *et al*. (2015) [19], and Basheva *et al*. (2015) [20]). The goal of these studies was mainly to explore the role of the rearrangements in the genetic structuring of populations (study of speciation models). However, in some studies carried out in mice ([21]; [22]; cited by Hulten (2011) [18]), a tendency for the frequency of chiasmata to be increased within the interstitial segment was also reported in carriers of reciprocal translocations.

To the best of our knowledge, a global analysis of the impact of chromosomal rearrangements on the course of meiosis has never before been carried out in livestock species. In the present paper, synapsis, recombination and interference were studied using cytological approaches in three boars carrying different chromosomal rearrangements involving *Sus scrofa* chromosomes 3 (SSC3) and 4 (SSC4). We examined whether the rearrangements modified the recombination landscapes and interference on these chromosomes. We were particularly interested in checking whether the breakpoints could represent a barrier to transmission of the interference signal. We hypothesized that the DNA sequence transition on rearranged chromosomes should slightly alter transmission of the interference signal in the translocation homozygotes.

## Materials and Methods

### Animals

Founder boars carrying original chromosomal rearrangements were recruited from the national program for the systematic control of young pedigree boars destined for artificial insemination centers [23]. One carried a balanced reciprocal translocation t(3;4)(p1.3;q1.5), and another carried a pericentric inversion inv(4)(p1.4;q2.3). Both were of the Large White breed. Sperm samples were cryopreserved at the INRA center of Rouillé (France). Matings between these two boars and normal sows (with normal karyotypes) of the same breed were carried out to produce F1 pigs heterozygote for these chromosomal rearrangements. Crosses between F1 pigs were then made to produce individuals with different karyotypes: one male heterozygote for the translocation (T34he), one male homozygote for the translocation (T34ho), and one male heterozygote for the two rearrangements simultaneously (T34Inv). Three boars (two Large White and one Meishan) with normal karyotypes were also selected as controls.

The experimental animals were bred in individual 6m^2^ boxes. The litter consisted of a thick (about 30 cm) layer of wood chips, renewed on a monthly basis. Animals had free access to water and were fed twice a day.

Testicular samples were collected by surgical hemi-castration. Pre-anesthesia (intramuscular injection of Ketamine, 10 mg/kg; VIRBAC, Carros, France) was followed by an inhalation anesthesia (isoflurane; VIRBAC, Carros, France). Post-operative follow-up was carried out in a recovery room adjacent to the operating facility. The animals were monitored until their awakening that occurred within 15 minutes after the operation. Castration was minimally invasive. Pain was relieved by an intramuscular injection of Finadyne (2 ml/50 kg; MSD Santé Animale, Beaucouze, France) which complemented the pre-anesthetic analgesic action. First getting up and the first meal were monitored and the healing process was controlled every day for 2 weeks.

Histopathological analyses were carried out as described by Barasc *et al*. (2014) [24]. These analyses did not reveal any alteration and the seminal parameters of the boars (concentration, mobility, and morphological parameters) were within the normal limits.

According to the European Directive 2010/63/EU on the protection of animals used for scientific purposes, this study was approved by the Ethics Committee for Animal Experimentation of the Poitou Charentes region (France) (CE2012-2), under the agreement number A-17-661.

### Immunocytology

Meiotic cells were prepared as previously described by Massip *et al*. (2010) [25]. The synaptonemal complex proteins 3 (SCP3) and 1 (SCP1), MutL homolog 1 protein (MLH1) and centromeres were detected using the following primary antibodies: rabbit anti-SCP3 (1:1000; ABCAM, Cambridge, UK), rabbit anti-SCP1 (2:1000; ABCAM, Cambridge, UK), mouse anti-MLH1 (2:100; Becton Dickinson, Franklin Lakes, NJ), Human anti-kinetochore (1:100; Antibodies Incorporated, Davis), respectively, and prepared in a solution of PBT (PBS +0.16% BSA +0.1% Tween). The secondary antibodies consisted of Alexa 594 conjugated donkey anti-rabbit (1:100, Molecular Probes), Alexa 488 conjugated goat anti-mouse (1:100, Molecular Probes, Eugene, OR, USA), and AMCA conjugated donkey anti-human (1:100, Jackson Immunoresearch, West Grove, PA, USA). The γH2AX protein was detected by carrying out a complementary experiment without MLH1 antibody but with mouse anti-γH2AX (ABCAM, Cambridge, UK) and Alexa 488 conjugated goat anti-mouse (1:100, Molecular Probes, Eugene, OR, USA) antibodies. γH2AX is considered as a marker of unsynapsed chromatin (transcriptionally silenced chromosome regions—Turner *et al*. (2005) [26])

### Fluorescent *in situ* hybridization (FISH)

BAC (bacterial artificial chromosome) probes were hybridized on the same slides according to Mary *et al*. (2014) [8]. These BAC probes were selected in the telomeric regions of the chromosomes [27] and were obtained from the Biological Resources Center-GADIE (http://www-crb.jouy.inra.fr/) [28].

Different combinations of probes were used:

1. A combination of three probes (one located on the telomeric region of the p-arm of chromosome 3 (526E5), and two probes located on the telomeric regions of the p-arm (100D4) and q-arm (330C8) of chromosome 4, respectively) was used to identify each arm of the quadrivalents in the spermatocytes of boars T34he and T34Inv.
2. In the case of boar T34he, 3 additional BAC probes located on the telomeric regions of chromosomes 2 (370D12), 8 (277F7) and 9 (736D9) were hybridized on the same cells. These 3 chromosomes (pairs) were not involved in the rearrangements, but are comparable in size and structure (position of the centromere) to SSC3 and SSC4, and were considered as internal controls.
3. A combination of two probes (one located on the telomeric region of the q-arm of SSC3 (639G2), and one located on the telomeric region of the p-arm of SSC4 (100D4)) was used to identify the two bivalents in the spermatocytes of boar T34ho.
4. A complementary experiment was carried out on one normal Large White boar (normal karyotype) for which all bivalents were identified using 21 BACs probes, as already described by Mary *et al*. (2014) [8]. The results obtained for this boar were pooled with those obtained in the previous study by Mary *et al*. (2014) [8] for two other normal boars.

Probes were labeled with biotin or digoxygenin using the bioprime labeling system (Invitrogen) and revealed using Alexa 594 conjugated to Streptavidin (Molecular Probes, Eugene, OR, USA) and FITC conjugated mouse anti-digoxygenin antibodies (Sigma, St Louis, MO).

### Image and Statistical Analyses

Spermatocytes were captured using the Cytovision FISH imaging system (Leica Microsystems, Nanterre, France). The images obtained after the FISH experiments were analyzed using MicroMeasure 3.3 software [29] to determine the physical length of each SC, and the relative positions of the centromere and CO (relative positions expressed as percentages of the total SC length). For it, all SC axes were drawn and the MLH1/centromeres foci were localized. For boars T34he and T34Inv, SC corresponding to the chromatin of SSC3 or SSC4 were measured, For T34ho, the bivalents formed by the translocated der(3) and der(4) chromosomes (i.e., bivalents formed by the SSC3 and SSC4 chromatin) were measured.

It was difficult (for T34he and T34Inv) or even impossible (for T34ho) to accurately map the breakpoints in the spermatocytes. Therefore, their positions were systematically determined using the relative “breakpoint to centromere” and “breakpoint to telomere” distances measured on derivative metaphasic GTG-banded chromosomes. The same relative distances were applied to predict the breakpoint locations on the spermatocyte chromosomes.

The frequency distributions of the inter-CO distances were fitted to the gamma model as explained in Broman and Weber (2000) [30]. Maximum likelihood estimates for the ν parameter of the gamma function were obtained using the free online Wessa software [31].

To determine whether the interference acted across the breakpoints, the relationship (Pearson correlation coefficient) between i) the distances between the breakpoint and the nearest MLH1 focus on the left side ([d (L)]), and ii) the distances between the breakpoint and the nearest MLH1 focus on the right side ([d (R)]), were analyzed using data from SC with at least one MLH1 signal on both sides of the breakpoint, as suggested by Colombo and Jones (1997) [6].

The strength of interference was also assessed in the T34ho boar by using a "coefficient of coincidence analysis" (as explained for instance in Fung *et al*. (2004) [32], and Libuda *et al*. (2013) [12]). The chromosomes were divided into three intervals of equal sizes and the MLH1 foci were allocated to these intervals (the amount of data available did not allow us to consider more than three intervals on the chromosomes of the T34ho boar). For each specified pair of intervals tested, the coefficient of coincidence corresponded to the ratio between the observed (O) number of MLH1 foci occurring in both intervals and the expected (E) number if foci occurred independently in the two intervals (that is, absence of interference). The interference strength (I) was calculated as (1 –O/E). The “expected” number of chromosomes with MLH1 foci occurring in both of a given pair of intervals (X and Y) was calculated as: E = (measured frequency of chromosomes exhibiting MLH1 foci in interval X) x (measured frequency of chromosomes exhibiting MLH1 foci in interval Y) x (total number of chromosomes examined).

The number of MLH1 foci and the length of SC (per chromosome and per cell) obtained for the three normal boars were pooled and compared with those obtained for boars T34he, T34ho and T34Inv, using a non-parametric Mann—Whitney U test. The MLH1 distributions on the SSC3 and SSC4 chromosomes in the different boars were compared by Kolmogorov-Smirnov (KS) test. This KS test was also used to assess whether the inter-CO distances distributions were well fitted by gamma distributions. R software was used for the statistical analyses. Due to the strong inter-individual and inter-cell (within the same individual) variation in recombination rates and SC lengths, well documented in humans [33] and pigs [8,34], P values < 0.01 were considered statistically significant for inter-individual comparisons.

## Results

At least 50 pachytene spermatocytes were studied for each boar carrying a chromosomal rearrangement (Table 1). One hundred and fifty one cells from one boar with a normal karyotype were also analyzed. The results from this latter boar were pooled with those already obtained for two other normal boars [8]. In total, 264 cells from 3 normal individuals were used as control (Table 1).

**Table 1:** Number of spermatocytes analyzed, mean MLH1 foci numbers and relative SC length per spermatocyte.

		Mean number of MLH1 foci per spermatocyte (±SE)				Mean relative SC length (%) per spermatocyte (±SD)^b^		
Individuals	No. of cells^a^	All autosomes	SSC3^c^	SSC4^c^	SSC3+4^c^	SSC3^c^	SSC4^c^	SSC3+4^c^
Controls	264	31.86 (0.18)	2.02 (0.04)	1.85 (0.03)	3.88 (0.06)	7.01 (1.04)	5.18 (0.58)	12.19 (1.20)
T34he	63	31.10 (0.54)	2.00 (0.07)	1.83 (0.06)		7.04 (1.10)	6.02 (1.19)**	
T34ho	80	30.43 (0.35)**			3.83 (0.10)			12.33 (1.35)
T34Inv	54	31.41 (0.37)	1.94 (0.08)	1.76 (0.06)		7.45 (0.93)*	5.94 (0.89)**	

^a^ number of spermatocytes analyzed by FISH (p and q arms identified for SSC3 and SSC4).

^b^ Percent of total autosomal SC length.

^c^ Chromatin corresponding to *Sus scrofa* chromosome 3 (SSC3), SSC4 or both (SSC3+4).

* P<0.01;

** P<0.001 compared to controls.

The length of SC, the number and the distribution of MLH1 foci for chromosomes SSC2, SSC8 and SSC9 (considered as control chromosomes) in boar T34he were not significantly different from the values obtained for the control boars (P>0.01; S1 Table).

The breakpoint positions on the der(3) and der(4) chromosomes were estimated using measures made on derivative metaphasic GTG-banded chromosomes (see the “Materials and Methods” section). The breakpoints were located at a distance of 70% and 30% of the total chromosome length (starting from the extremity of the q-arms) for chromosomes der(3) and der(4), respectively.

### Rearranged chromosomes and synapsis

**Fig 1:**
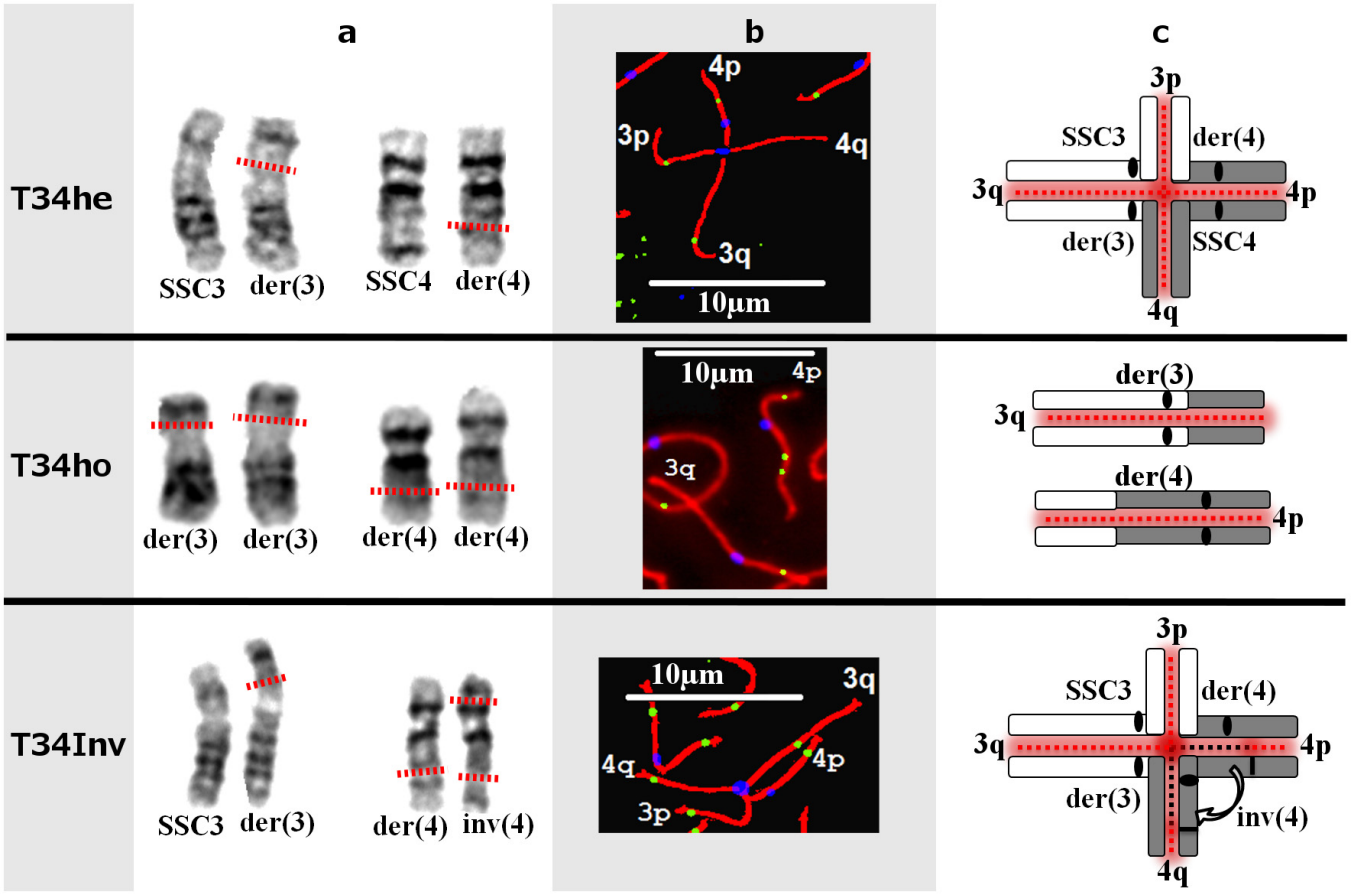
GTG banding and synapsis at meiosis-I for each chromosomal rearrangement. (a) GTG banding of the chromosomes involved in the rearrangements. The dotted lines in red represent the breakpoints locations. (b) Identification of chromosome arms on spermatocytes after FISH of BAC clones (more details in S1 Fig) and immunolocalization of SCP1-SCP3 (red), MLH1 (green) and kinetochores (blue). (c) Schematic representation of the synapsis between chromosomes. The dotted lines in red represent the normal synapsis, the dotted line in black the heterosynapsis, the white rectangles represent the SSC3 chromatin and the grey rectangles the SSC4 chromatin, the black ovals represent the centromeres and the black lines on the T34Inv represent the breakpoints.

Immunolocalization of the meiotic proteins, coupled to FISH, was used to identify and orient the SC for normal and rearranged chromosomes 3 and 4 (S1 Fig). Analysis of the spermatocytes-I in the three boars carrying chromosomal rearrangements revealed three different pairing configurations. In boar T34he, heterozygote for the t(3;4) reciprocal translocation, quadrivalents combining one copy of SSC3, one copy of SSC4, as well as one der(3) and one der(4) chromosomes were observed (Fig 1). This configuration allowed pairing between the homologous regions of the different chromosomes. In boar T34ho, homozygote for the same translocation, two pairs of homologous "neo-chromosomes" were observed (Fig 1). The telomeric part of the p-arms of the der(3) chromosomes originated from the distal part of the q-arms of normal SSC4. Reciprocally, the telomeric part of the q-arms of the der(4) chromosomes originated from the distal part of the p-arms of normal SSC3. These two pairs of "neo-chromosomes" (one pair of der(3) and one pair of der(4) chromosomes) normally paired as bivalents (Fig 1c). In boar T34Inv, heterozygote for both rearrangements (translocation and inversion), quadrivalents were also observed, as for boar T34he, but in this case the inv(4) chromosome was upside down (as compared with SSC4 in boar T34he; Fig 1c). Homologous pairing occurred in the telomeric regions of chromosome inv(4) (distal to the breakpoints), whereas heterosynapsis was observed in the centromeric part of this chromosome (between the two breakpoints). Such heterosynapsis had already been observed in 90% of the spermatocytes in another boar carrying the same pericentric inversion of chromosome 4 [25].

To identify putative synaptic defects, immunolocalization of the γH2AX protein was carried out on spermatocytes from the 3 boars. No γH2AX signal could be detected, except on the XY body (S2 Fig).

### Length of SC

The physical lengths of SC (in μm) are known to vary greatly between cells from the same individual, or between individuals. Conversely, the relative lengths (in %) between the same chromosomes from different boars are generally comparable [8]. The impact of chromosomal rearrangements on the length of SC was determined by comparing the relative lengths of the chromosomes involved in the rearrangements with the relative lengths of the same chromosomes in control individuals with normal karyotypes (Table 1).

For boar T34he, a significant increase in relative length was only observed for chromosome 4 (relative length of the SC associating the normal SSC4 with the homologous parts of der(3) and der(4) chromosomes—see Fig 1c, as compared to the length of the SSC4 bivalents in the control boars; P<0.001 –Table 1). For boar T34Inv, a significant increase was observed (P<0.01 –Table 1) for chromosomes 3 (relative length of the SC associating the normal SSC3 with the homologous parts of der(3) and der(4) chromosomes, see Fig 1c) and 4 (relative length of the SC associating the inv(4) with the homologous parts of der(3) and der(4) chromosomes, see Fig 1c). The comparison was more difficult for boar T34ho because the SSC3 chromatin could not be distinguished from the SSC4 chromatin on the derivative chromosomes (the limit between the SSC3 and SSC4 chromatin on the derivative chromosomes could not be accurately determined). Conversely, comparison of the total length of both derivative chromosomes and the cumulative length of the normal SSC3+SSC4 in control boars was possible and relevant. As indicated in Table 1, no significant difference was observed.

### Number of MLH1 foci

The average number of MLH1 foci per spermatocyte for boars T34he, T34ho and T34Inv was 31.10, 30.43 and 31.41, respectively (Table 1). These results did not differ significantly from those of the control boars (31.86 on average) except for boar T34ho (P<0.001). However, the magnitude of the difference between these two values (30.43 vs 31.86) remained low as compared with the range observed in normal boars (30.2 to 37.3) [8].

For boars T34he and T34Inv, the recombination rates observed for chromosomes 3 and 4 were comparable to those obtained for the controls (the observed differences were not statistically significant–Table 1).

The cumulative number of MLH1 foci on the two bivalents corresponding to the der(3) and der(4) chromosomes in boar T34ho was not statistically different from the cumulative number of MLH1 foci on SSC3+SSC4 in control boars (Table 1). Thus, the smaller number of MLH1 foci per cell observed in this boar probably reflected the natural, biological variability between individuals.

### MLH1 foci distributions

#### Boar T34he

**Fig 2:**
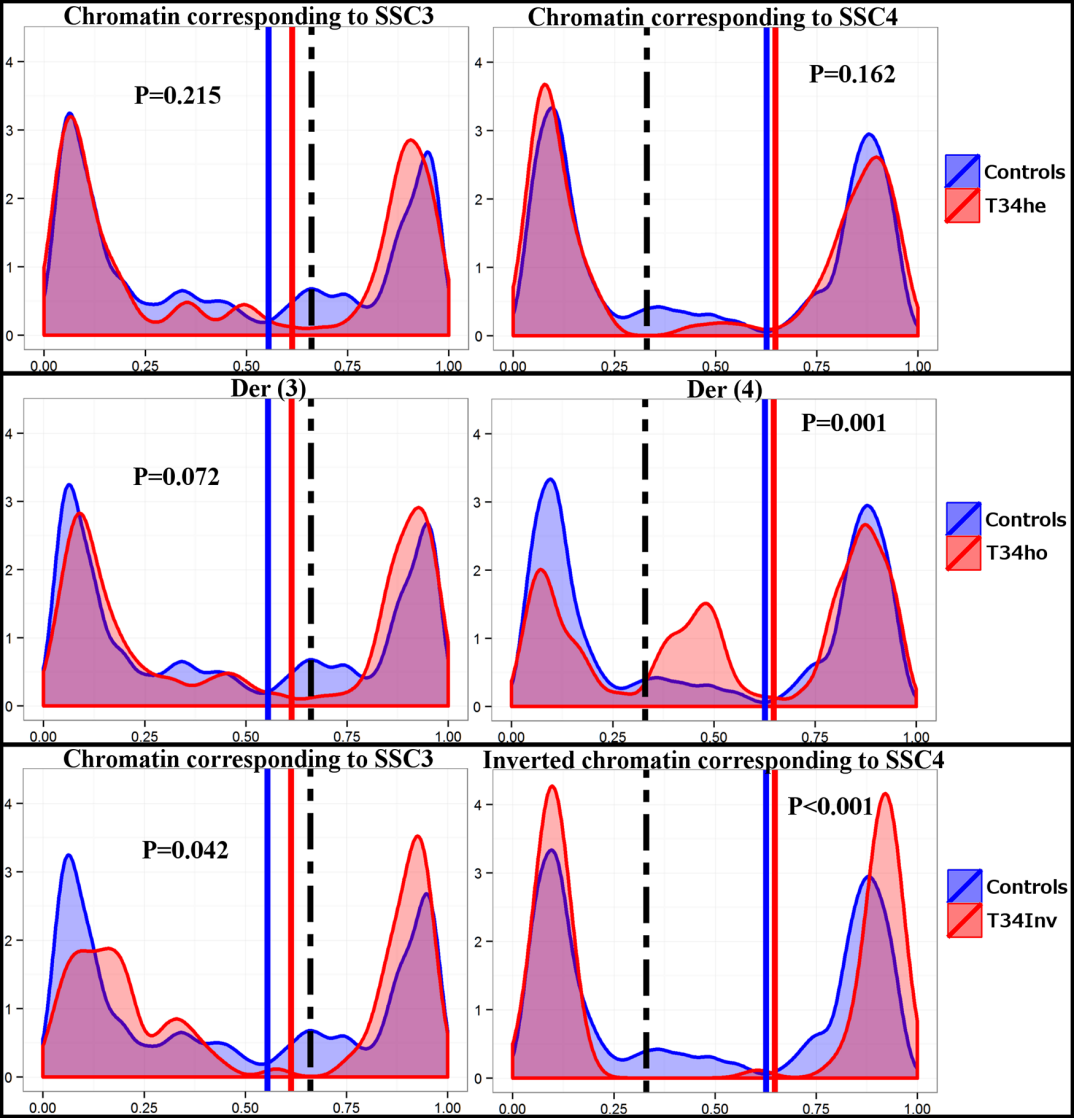
Comparison of the MLH1 distributions between rearranged chromosomes and controls. The y-axis indicates the frequency of the MLH1 signals along the SCs on the controls (in blue) and on the rearranged chromosomes (in red). The x-axis represents the length of the SCs in percent from the q (left) arm to the p (right) arm. The vertical lines in bold represent the centromeres and the dotted line represents the estimated position of the breakpoints on the translocated chromosomes.

The impact of the rearrangement on the location of CO was studied by determining the distributions of MLH1 foci along the chromosomes involved in the quadrivalent (chromosome 3 and chromosome 4, Fig 2), and comparing them with the control. No significant difference was noted, despite the slight decrease in the recombination rate in the vicinity of the breakpoints (estimated location of the breakpoints).

#### Boar T34Inv

As illustrated in Fig 2, the distribution of MLH1 foci for chromosome 4 was strongly and significantly modified (P<0.001) as compared to the controls. In boar T34Inv, almost no MLH1 signal could be detected in the central, heterosynaptic region (heterosynapsis between the inverted chromosome 4 and the der(3) and der(4) chromosomes–Fig 1). This would indicate an abolition of recombination in the region where the paired chromosomes were non-homologous, and a transfer of the CO towards the terminal ends of the chromosome. We also noticed a difference (less pronounced, P<0.05) between boar T34Inv and the controls for chromosome 3 (Fig 2). In this case, a decreased recombination rate was noted in the vicinity of the breakpoint, as for boar T34he.

#### Boar T34ho

The distributions of MLH1 foci along the bivalents corresponding to the paired der(3) and paired der(4) chromosomes were analyzed and compared to the controls (SSC3 and SSC4 bivalents in individuals with normal karyotypes). No significant difference (p>0.07) was observed for der(3): one peak on each extremity of the chromosome, and a slight decrease of the number of MLH1 foci in the vicinity of the breakpoint were noted. Conversely, the distribution for the der(4) chromosome differed significantly from that observed in the controls (p<0.001). The same peak was observed on the extremity of the p-arms. However, two distinct peaks were noted on the q-arms, which carried the breakpoint (the translocated chromosomal fragment), each peak being of a smaller magnitude than the single peak observed in the controls (Fig 2). The region between the two peaks, approximately in the middle of the q-arms, should correspond to the breakpoint (transition between SSC4 chromatin and SSC3 chromatin on the der(4) chromosomes).

### Distributions of inter-foci distances

**Fig 3:**
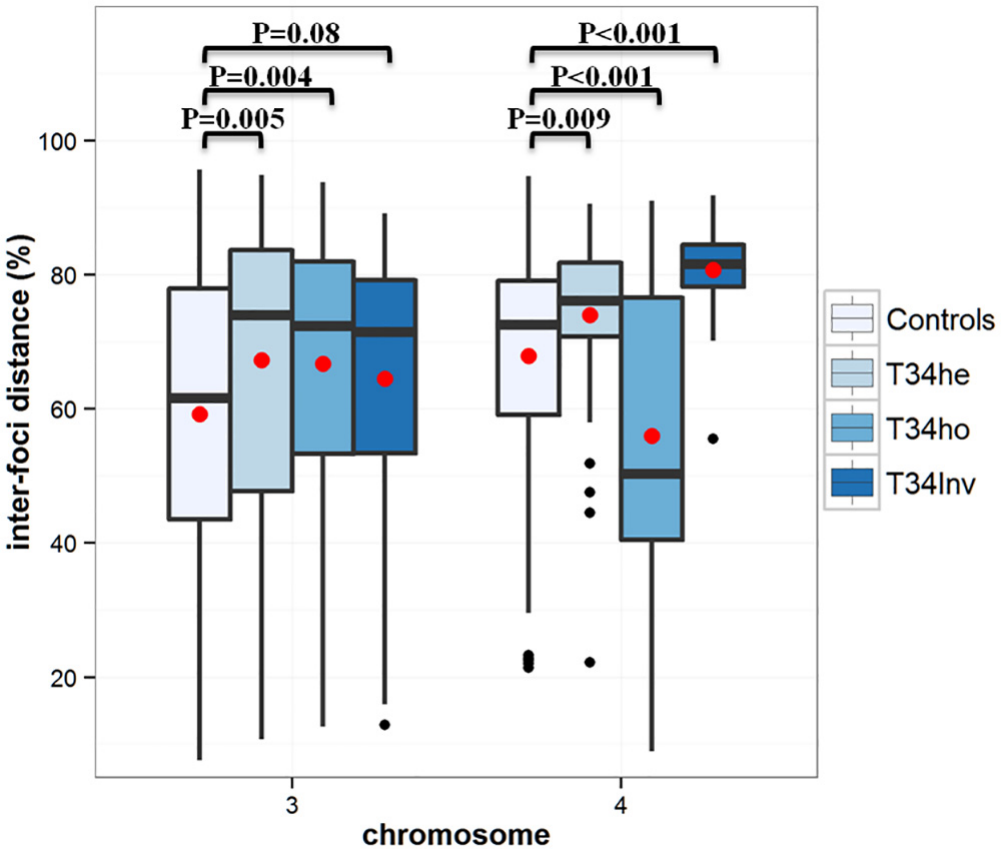
Box-plot diagrams showing the inter-foci distances (in % SC) measured for chromosomes 3 and 4 in the different boars. The black horizontal bars and the red points represent the median and the mean values, respectively.

The distributions of the inter-foci (MLH1) distances obtained for boars T34he, T34ho and T34Inv, were fitted to gamma functions. The quality of fit was highly variable: good or relatively correct for chromosome 4, moderate, or even poor for chromosome 3 (S3 Fig). Therefore, only the mean and median inter-foci distances (between COs in % of SC length) will be presented and discussed (Fig 3). For chromosome 3, the means for boars T34he and T34ho (67.2, and 66.8, respectively) were significantly higher (P<0.01) than the value obtained for the control boars (59.2). This was not the case for T34Inv (64.5). For chromosome 4, the means for boars T34he, T34ho and T34Inv (74.0, 56.0 and 80.7, respectively) were significantly different (P<0.01) from the control (67.9). The average inter-foci distance was significantly lower than the control only for chromosome der(4) of the T34ho boar.

### Breakpoints, barriers to interference?

Observations in several species, including the pig, have indicated that interference might act through the centromeres [8,35]. One of our objectives was to investigate whether or not the interference also acted through the breakpoints on rearranged chromosomes.

**Fig 4:**
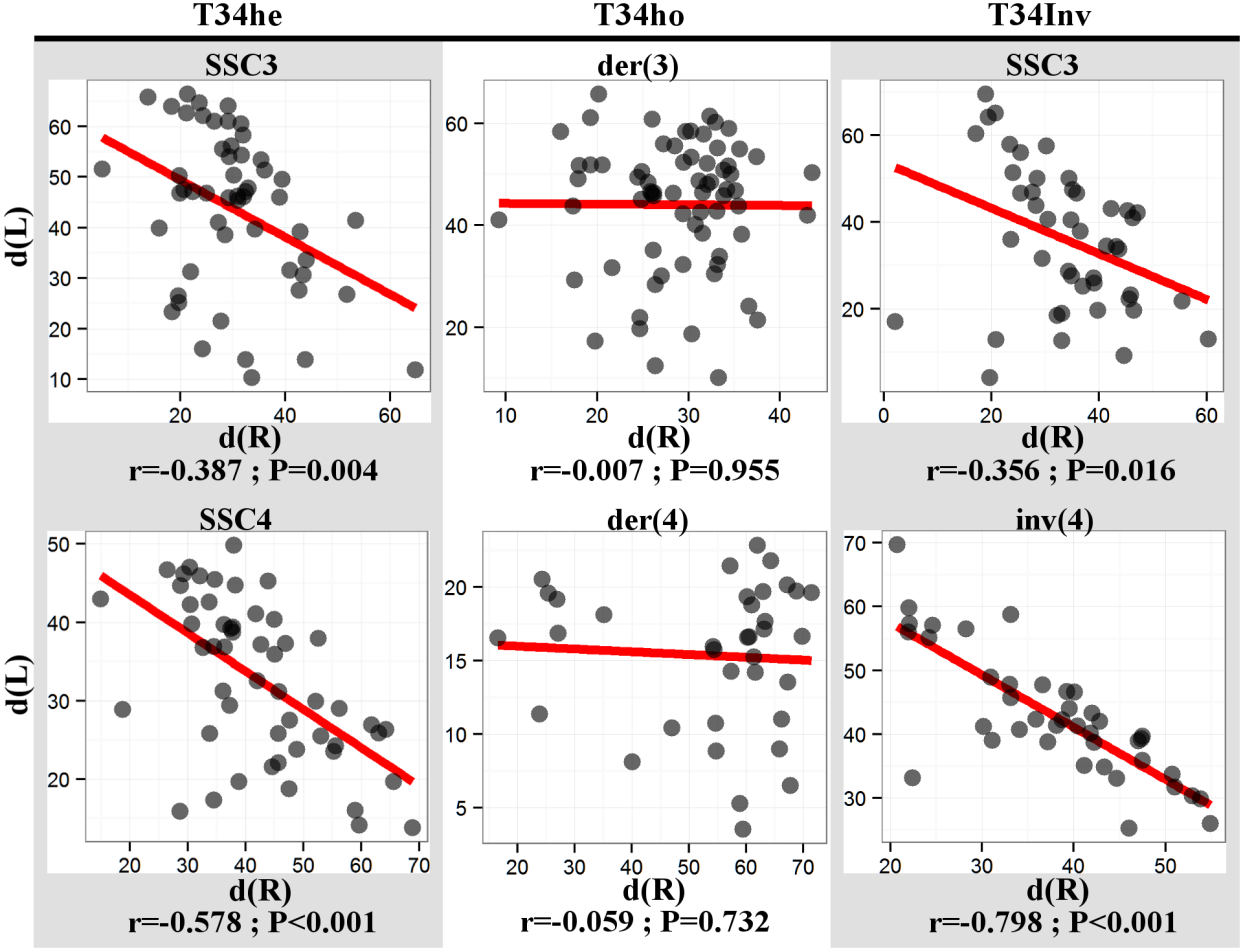
Relationship between the two distances from breakpoint to the nearest CO on the left [d(L)] and right [d(R)] sides of chromosomes that have at least one CO on each side. The results of the correlation analyses are indicated for each boar. The distances are expressed as percentage of the SC length.

The correlation coefficients between the two distances from breakpoint to the nearest CO on the left [d(L)] and right [d(R)] sides of the breakpoint (see Material and Methods section) computed for chromosomes 3 and 4 in boar T34he were negative and significantly different from zero (r = -0.387 and -0.578, respectively; Fig 4). This was also the case for the correlation coefficient computed for chromosome 3 from boar T34Inv (r = -0.356). These results suggest that, when a CO comes close to the breakpoint, the CO on the other side of the breakpoint is pushed away. The CO were not distributed independently on both sides of the breakpoints in the (3;4) translocation heterozygotes, i.e., the interference seemed to act through the breakpoints.

Analyses carried out on chromosome 4 from boar T34Inv also revealed a strongly negative correlation coefficient (r = -0.798). This result indicated that, despite the relatively large distance between the MLH1 foci on chromosome 4, interference was still present. This interference was not impeded by the breakpoint or by the heterosynapsis in the heterologous pairing region.

Conversely, the correlation coefficients computed for the der(3) and der(4) chromosomes from boar T34ho were both close to zero (-0.007 and -0.059, respectively). In that particular case (homozygous individual), the breakpoints seemed to alter the transmission of the interference signal.

Complementary analyses were therefore carried out to further investigate this observation.

First, a comparable correlation analysis was carried out for SSC3 and SSC4 normal bivalents (considered as “control chromosomes”) in the control boars using the inferred would-be breakpoint as reference. The obtained correlation coefficients were slightly negative (-0.278 and -0.177 for SSC3 and SSC4, respectively), but significantly different from zero, suggesting that a (weak) interference signal was transmitted through that particular chromosomal region in normal chromosomes (S4 Fig).

**Fig 5:**
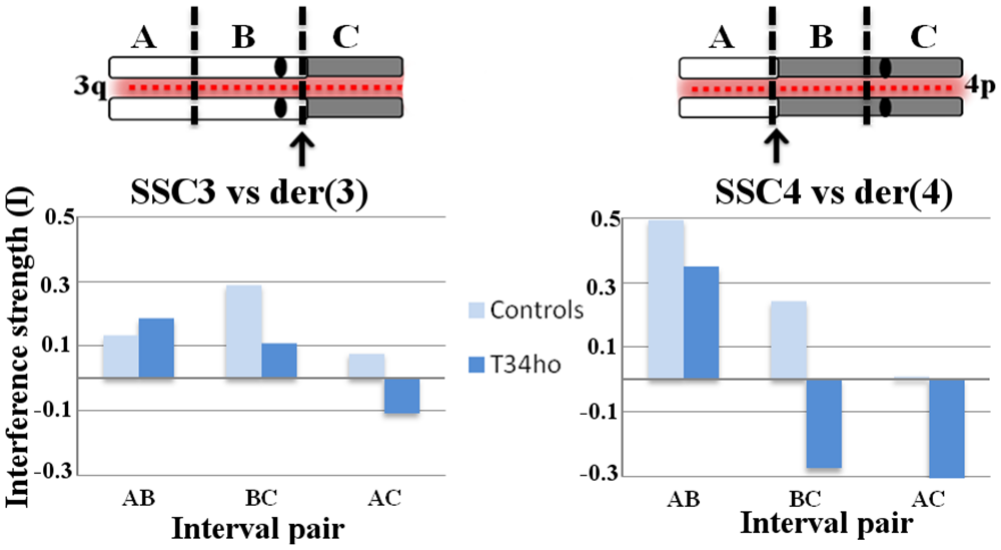
Graph of interference strength values (see Methods) for the indicated interval pairs in boars T34ho and controls. The arrows represent the putative location of the breakpoints on the der(3) and der(4) chromosomes.

Secondly, the strength of interference was assessed in this T34ho boar (as well as in control boars) using a “coefficient of coincidence analysis” (see Material and Methods section). As shown in Fig 5, the der(3) and der(4) chromosomes (respectively SSC3 and SSC4 in control boars) were divided into three regions of comparable sizes. For the der(3) chromosomes, the limit between the B and C segments was chosen in the region where the breakpoints occurred (limit between A and B for der(4) chromosomes). For chromosome der(3) (respectively SSC3), a positive interference was estimated for the adjacent AB pair of intervals (for T34ho and control boars; Fig 5). The level of interference was logically reduced for the more distant AC pair of intervals (interference was even “negative” for the T34ho boar, indicating that a chromosome with a focus in one end-interval has an increased likelihood of having a second focus in the opposite end-interval). For the other adjacent BC pair of intervals, interference was still present in the control boars (even higher than the interference for the AB pair), while it was almost halved in the T34ho boar, as compared with the interference over the AB segment. These results seemed to confirm that the SSC3 to SSC4 chromatin transition on the der(3) chromosomes (i.e. occurrence of the breakpoint) would alter the transmission of the interference signal. The results obtained for SSC4 chromosomes in control boars were also logical: positive interference for adjacent intervals AB and BC, and lack of interference for the most distant pair of intervals (AC). The results were more intriguing for the der(4) chromosomes (Fig 5). A positive interference was observed for the AB pair of intervals (a lower value was expected because of the breakpoint occurring in that segment), while it was negative for the BC pair of intervals (a positive interference was expected). As for der(3), the interference level for the more distant AC pair of intervals was reduced for the T34ho boar as compared to control boars.

## Discussion

The mechanisms that control the number and distribution of CO (obligatory CO, homeostasis, interference) involve communication along the chromosomes. We do not know the exact nature of the signal or the medium of this communication. However, we can hypothesize that the signals propagate along the SC, or along the chromatin (DNA and/or associated proteins), or both. Several models have been proposed to explain the ways in which CO can be regulated within the cell. Some authors suggest that the number of CO and the interference are controlled by different mechanisms, whereas others propose a common basis [5,18,36]. This question has not been fully answered to date and was therefore investigated here.

All three boars in the present study carried various chromosomal rearrangements but presented normal semen parameters. This suggests that meiosis followed a normal course in these individuals. In boar T34he, heterozygote for the (3;4) translocation, this could be explained by the formation of a quadrivalent, allowing correct synapsis between homologous chromosomal regions. In boar T34ho, homozygote for the (3;4) translocation, normal synapsis also occurred between the "neo" (derivative) chromosomes (formation of two bivalents). Quadrivalents with complete synapsis were even observed in the relatively complex rearrangement carried by boar T34Inv. These results were consistent with those obtained by Massip *et al*. (2010) [25] for another boar carrying the same pericentric inversion (heterosynapsis of the inverted region, also described in Human cases [37,38]). However, in 10% of the spermatocytes from this latter boar, an inversion loop was also observed [25], which was not the case in our study. This could be explained by the formation of the quadrivalent in boar T34Inv (steric hindrance of the quadrivalent in the vicinity of the breakpoints preventing the formation of a nearby loop). Moreover, inversion loops can also be resolved by synaptic adjustment to produce a straight SC during the meiotic-I prophase [39].

The immunofluorescence corresponding to SCP1/SCP3 antibodies was apparently normal in all our preparations. However, this does not definitely prove that the SC were absolutely normal in all situations. Indeed, in a previous work carried out in *C*. *elegans*, Libuda *et al* (2013) [12] showed that apparently normal immunofluorescent patterns could be obtained when part (60–70%) of the SC central region proteins (Syp-1) were depleted (using RNA interference). Nevertheless, as no γH2AX signal was observed, major pairing defects could reasonably be excluded in our cases.

Because of the absence of a (major) pairing defect in our three boars, we were able to study the impact of the chromosomal rearrangements on the number and distribution of CO along the SC formed by the SSC3 and SSC4 chromatin. Comparable studies have been carried out in the past in various organisms. As summarized in the introduction, most of these studies revealed a major impact of chromosomal rearrangements on the recombination landscape. Nevertheless, relatively recent publications reported a quite moderate effect of autosome-autosome translocations on the recombination rates in Humans [40–42], which is more consistent with our own observations.

No modification in the number of CO on the chromosomes involved in the rearrangements was observed in our three boars, as compared to the control boars with normal karyotypes.

In boar T34he, the formation of a quadrivalent had little effect on the distribution of MLH1 signals. The observed modifications were relatively minor and restricted to the breakpoint regions (decreased number of MLH1 foci in the vicinity of the breakpoints–Fig 2). During the formation of the quadrivalent, steric hindrance could occur in the region where the eight chromatids join. This could locally prevent the access of proteins involved in the recombination process, or impair chromatin compaction. This could explain the slight increase in the SC length observed for the chromosomes involved in the rearrangement and, in addition, the slight decrease in number of MLH1 foci in the vicinity of the breakpoints. For boar T34he, we also observed an increase in the average inter-foci distances for chromosomes 3 and 4, which could be explained by the slight decrease of the number of MLH1 foci in the vicinity of the breakpoints. Still in boar T34he, we showed (Fig 4) that the interference signal propagated along the chromosomes (one normal chromosome 3 pairing with der(3) and der(4), as well as one normal chromosome 4 pairing with der(3) and der(4)—Fig 1c). The interference signal could propagate along the uninterrupted chromatin of the normal chromosomes (3 and 4), or along the SC.

In boar T34Inv we also observed an increase in the inter-foci distance for chromosome 3. However, this increase was smaller than that observed for the same chromosome in boar T34he, and not significant. We also observed a slight decrease in the number of MLH1 foci in the vicinity of the breakpoint for this chromosome (Fig 2). The interference signal propagated on chromosome 3 of T34Inv, as in T34he (Fig 4) and the control (S4 Fig). On chromosome 4 from this boar T34Inv (inverted chromosome 4, associated with der(3) and der(4) on the quadrivalent–Fig 1c), the distribution of MLH1 foci was strongly modified. A lack of MLH1 signal had already been noted in the central heterosynaptic region (Fig 2). The opposite chromatids in that region did not show sequence homology. The lack of CO in that region could be due to the impossibility for the invading single-strand DNA to find any homologous sequence. It is interesting to note that the decreased recombination rate in the central region is compensated by an increase in the distal regions, where the sequences are homologous. This visible recombination transfer is consistent with the comparable global recombination rate for chromosome 4, between T34Inv and the controls (Table 1). Such an intrachromosomal “compensation effect” had already been reported in *Arabidopsis* [16] and *C*. *elegans* [13,17]. The lack of MLH1 signals in the heterosynaptic region (which represents about 75% of the SC length [25]) led to a strong increase in the inter-foci distance on this SC (Fig 3). As shown by the strongly negative correlation between [d (L)] and [d (R)] (Fig 4), interference was still present, despite the large distance and the heterosynapsis of the region. The signal(s) regulating the number of CO and interference would be able to propagate over long distances (the average distance between adjacent MLH1 foci represented about 80% of the SC length), along the chromatin of the inv(4) chromosome, or along the SC. This is consistent with the results of Hillers and Villeneuve (2003) [11] who demonstrated, using chromosome fusions in *C*. *elegans*, that regulation can operate over large physical distances, encompassing up to half the genome.

The impact of chromosomal inversions on recombination and interference has been studied occasionally in the past. Most studies used murine models and produced quite heterogeneous results. Gorlov and Borodin (1995) [43], for instance, showed that a large inversion had no effect on the global recombination rate. In an earlier study, the same authors had observed a reduction of recombination due to a decrease in the number of chiasmata in the inverted region [44]. In men, Kirkpatrick *et al*. (2012) [45] also observed a reduction of MLH1 foci in an inv(1) carrier. However, this decrease was mainly explained by the formation of unpaired bivalents. In mice oocytes, a decreased recombination rate in the heterosynaptic region was also compensated by an increase in the distal region [46].

In boar T34ho, homozygote for the (3;4) translocation, the distribution of MLH1 signals was only marginally modified for chromosome der(3), as compared with SSC3 in the control (Fig 2). We only observed a slight decrease in the number of MLH1 foci near the breakpoint, which could explain the increase in the average interfoci distance on this chromosome (Fig 3). Such a result was also obtained in the heterozygotes (T34he and T34Inv) and could be explained (see above). Interpretation for the homozygote is more difficult. Conversely, the distribution of MLH1 signals in boar T34ho was strongly modified for the der(4) chromosome (as compared with SSC4 in the controls): the inter-foci distance was reduced (Fig 3) and two recombination peaks occurred on the q-arms, instead of the single one classically observed in the telomeric region. In view of the homology and normal pairing of the two der(4) chromosomes, this result was relatively unexpected. Such an increase in the frequency of chiasmata localized within the interstitial segment has already been reported in Human studies [18], but mainly in translocation heterozygotes (not homozygotes).

The very low correlations between [d (L)] and [d (R)] for both der(3) and der(4) bivalents in this boar T34ho seemed to indicate that the interference on these chromosomes was attenuated. Interference appeared to be halted by the breakpoints, i.e. partially lost during the SSC3 chromatin—SSC4 chromatin transition (and vice versa) on these derived chromosomes. The presence of a single normal SC would therefore not be sufficient to correctly propagate the interference signal, suggesting that the information provided by the chromatin (DNA and/or associated proteins) is also crucial for that propagation. However, as observed in the control boars, the correlation coefficients estimated for normal chromosomes were also quite low. This could indicate that interference is normally weak or difficult to detect in that region, or with this method. The "coefficient of coincidence analysis" carried out for chromosomes der(3) seems to confirm an attenuation of the interference signal. Unfortunately, this was not the case for chromosome der(4). However, we showed that the recombination landscape was strongly modified on the der(4) q-arms, where the breakpoint occurred (one CO hotspot region in the telomeric part of normal SSC4, split into two hotspots on der(4) chromosomes, on both sides of the breakpoint, i.e. accumulation of CO on both sides of the breakpoint). This particular situation makes the analysis of the transmission of an interference signal quite uncertain. Otherwise, despite the possibly disturbed interference, the number of MLH1 signals on the derived chromosomes of boar T34ho was not modified. The original recombination rates in the chromatin regions that were displaced by the rearrangement seemed to be maintained. This would indicate that (i) the interference, and (ii) the number of MLH1 signals, are (at least partially) differentially regulated.

## Conclusion

In most mammalian species, the SC are longer and the recombination rates higher in females than in males. A positive correlation between the length of SC and the number of MLH1 foci has also been reported in humans [47], mouse [48] and pig [8]. This suggests that the SC is directly involved in the regulation of recombination rates, or that extension of chromosome axes locally occurs in response to CO designation [12]. The normal SC that formed for all chromosomes in our three boars could explain the normal MLH1 number per chromosome observed in these individuals, despite some distribution changes.

In the cases of quadrivalents (observed in T34he and T34Inv boars), the continuous SC and the presence of at least one normal (or inverted) chromatid per chromosome (i.e., chromatids formed by continuous SSC3 or SSC4 chromatin) seemed sufficient to allow a correct propagation of the interference signal. Conversely, in the cases of "neo-chromosomes" (T34ho), the occurrence of a SSC3 to SSC4 chromatin transition (i.e., each chromatid formed by SSC3 + SSC4 chromatin) could partly explain the apparent loss of interference signal. This relatively unexpected result should be confirmed by studying meiotic recombination and interference in other cases of translocation homozygotes.

Based on these results, we can consider that the DNA sequence and/or nature (structure) of the associated proteins would be an important regulator of interference. Epigenetic mechanisms could be involved. Indeed, epigenetic modifications of the chromatin are known to influence meiotic recombination. For instance, in *Arabidopsis thaliana* [49], loss of DNA methylation in the centromeric region has been associated with an increased recombination rate in that region. Moreover, this increase was compensated by a decrease in the other chromosomal regions, allowing a constant number of CO per chromosome to be maintained. To extend our analyses further, it would be interesting to relate the partial (and apparent) disruption of interference observed on the rearranged chromosomes of the homozygote with the characteristics of its epigenome.

## Supporting Information

S1 Fig Spermatocytes after immunolocalization of SCP1-SCP3 (red), MLH1 (green) and kinetochores (blue), as well as FISH of BAC clones. T34he: 526E5 (SSC3qter, purple), 100D4 (SSC4pter, yellow), 330C8 (SSC4qter, white), 370D12 (SSC2pter, purple), 277F7 (SSC8qter, white) and 736D9 (SSC9pter, yellow). T34ho: 526E5 (SSC3qter, yellow) and 100D4(SSC4pter, white). T34Inv: 526E5 (SSC3qter, purple), 100D4 (SSC4pter, yellow), 330C8 (SSC4qter, white).
(PDF)

S2 Fig Meiotic pairing analysis of pachytene cells. Identification of chromosome arms on spermatocytes after immunolocalization of SCP1-SCP3 (red), γH2AX (green) and kinetochores (blue). No γH2AX-positive region was observed, except on the XY-body.
(PDF)

S3 Fig Gamma probability distribution modeling of inter-foci distances. The best-fit gamma probability distribution curves generated from modeling the data (and the corresponding υ parameter) are overlaid on the histograms. The goodness of fit was assessed using the Kolmogorov-Smirnov test (P-values).
(PDF)

S4 Fig Relationship between the two distances from the inferred “would-be breakpoint” to the nearest CO on the left [d(L)] and right [d(R)] sides of chromosomes that have at least one CO on each side for control boars. For the two chromosomes, results of correlation analyses are indicated. The distances were expressed as percentage of the SC length.
(PDF)

S1 Table Number of spermatocytes analyzed, mean MLH1 foci numbers and relative SC length per spermatocytes, and MLH1 distribution comparison. (PDF)
