# Supplementary material for: Meiotic Recombination Analyses in Pigs Carrying Different Balanced Structural Chromosomal Rearrangements
Source: PLoS One. 2016 Apr 28;11(4):e0154635. doi: 10.1371/journal.pone.0154635 (PMC4849707; doi:10.1371/journal.pone.0154635)
Supplement: S1 Fig — T34he: 526E5 (SSC3qter, purple), 100D4 (SSC4pter, yellow), 330C8 (SSC4qter, white), 370D12 (SSC2pter, purple), 277F7 (SSC8qter, white) and 736D9 (SSC9pter, yellow). T34ho: 526E5 (SSC3qter, yellow) and 100D4(SSC4pter, white). T34Inv: 526E5 (SSC3qter, purple), 100D4 (SSC4pter, yellow), 330C8 (SSC4qter, white). (PDF) [file pone.0154635.s001.pdf]

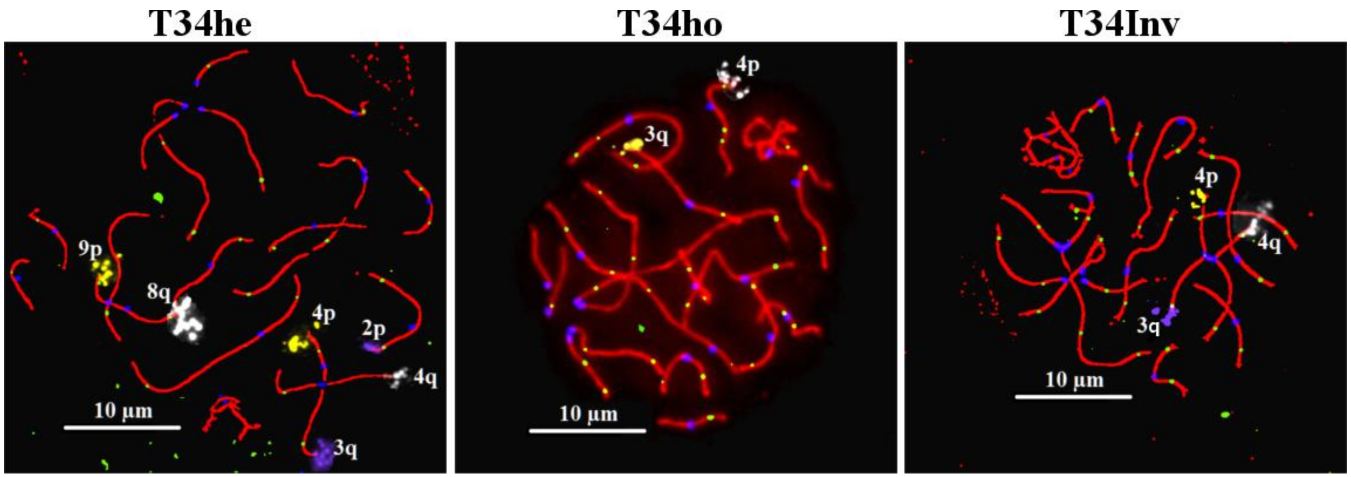

S1 Fig. Spermatocytes after immunolocalization of SCP1-SCP3 (red), MLH1 (green) and kinetochores (blue) and FISH of BAC clones.

-T34he: 526E5 (SSC3qter, purple), 100D4 (SSC4pter, yellow), 330C8 (SSC4qter, white), 370D12 (SSC2pter, purple), 277F7 (SSC8qter, white) and 736D9 (SSC9pter, yellow).

-T34ho: 526E5 (SSC3qter, yellow) and 100D4(SSC4pter, white).

-T34Inv: 526E5 (SSC3qter, purple), 100D4 (SSC4pter, yellow), 330C8 (SSC4qter, white).
