# Supplementary material for: Meiotic Recombination Analyses in Pigs Carrying Different Balanced Structural Chromosomal Rearrangements
Source: PLoS One. 2016 Apr 28;11(4):e0154635. doi: 10.1371/journal.pone.0154635 (PMC4849707; doi:10.1371/journal.pone.0154635)
Supplement: S2 Fig — Identification of chromosome arms on spermatocytes after immunolocalization of SCP1-SCP3 (red), γH2AX (green) and kinetochores (blue). No γH2AX-positive region was observed, except on the XY-body. (PDF) [file pone.0154635.s002.pdf]

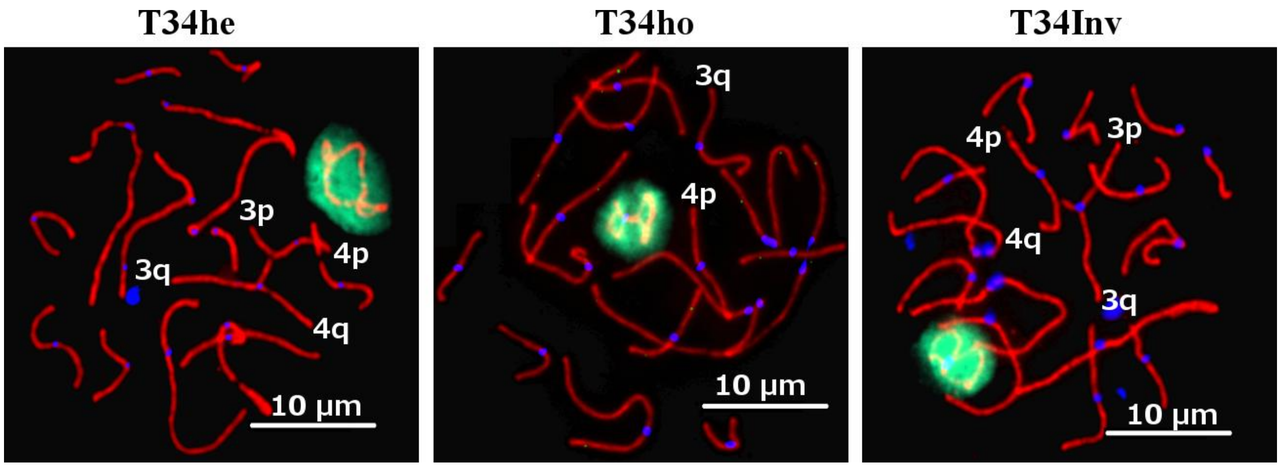

S2 Fig. Meiotic pairing analysis of pachytene cells. Identification of chromosome arms on spermatocytes after immunolocalization of SCP1-SCP3 (red), γH2AX (green) and kinetochores (blue). No γH2AX-positive region was observed, except on the XY-body.
