# Supplementary material for: Meiotic Recombination Analyses in Pigs Carrying Different Balanced Structural Chromosomal Rearrangements
Source: PLoS One. 2016 Apr 28;11(4):e0154635. doi: 10.1371/journal.pone.0154635 (PMC4849707; doi:10.1371/journal.pone.0154635)
Supplement: S3 Fig — The best-fit gamma probability distribution curves generated from modeling the data (and the corresponding υ parameter) are overlaid on the histograms. The goodness of fit was assessed using the Kolmogorov-Smirnov test (P-values). (PDF) [file pone.0154635.s003.pdf]

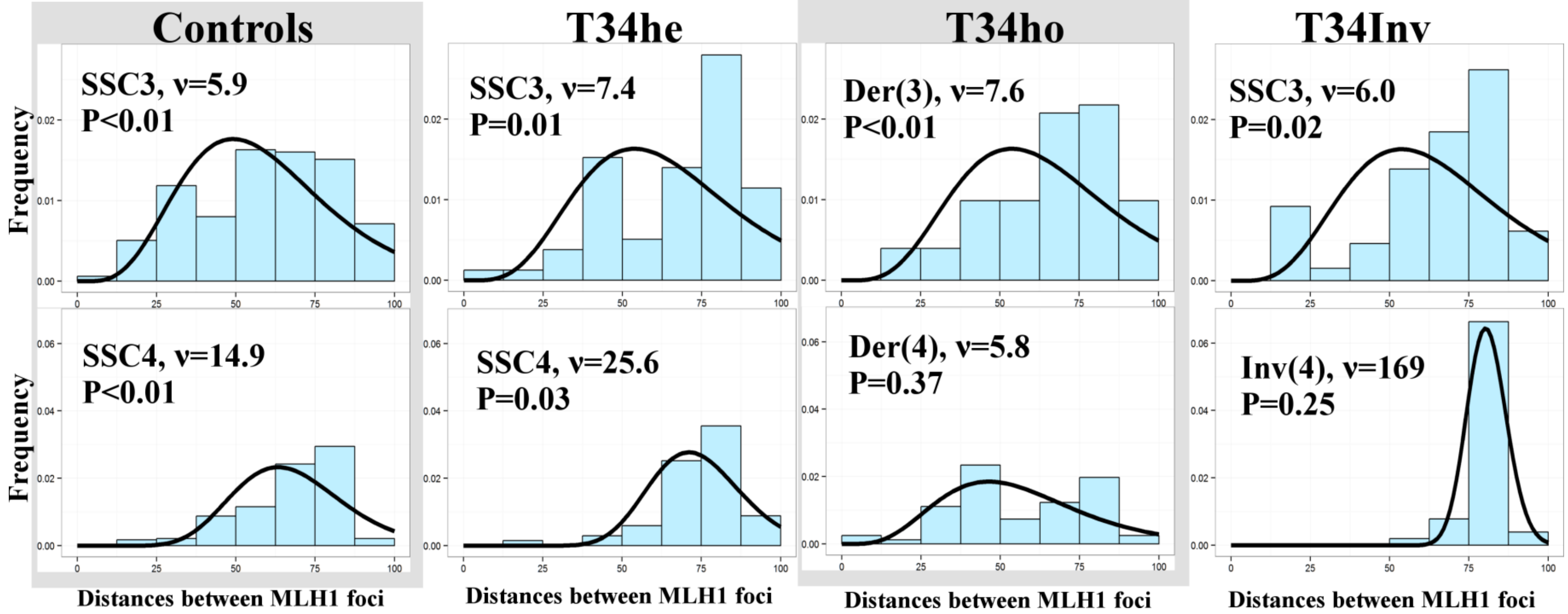

S3 Fig. Gamma probability distribution modeling of inter-foci distances. The best-fit gamma probability distribution curves generated from modeling the data (and the corresponding υ parameter) are overlaid on the histograms. The goodness of fit was assessed using the Kolmogorov-Smirnov test (P-values).
