# Supplementary material for: Meiotic Recombination Analyses in Pigs Carrying Different Balanced Structural Chromosomal Rearrangements
Source: PLoS One. 2016 Apr 28;11(4):e0154635. doi: 10.1371/journal.pone.0154635 (PMC4849707; doi:10.1371/journal.pone.0154635)
Supplement: S4 Fig — For the two chromosomes, results of correlation analyses are indicated. The distances were expressed as percentage of the SC length. (PDF) [file pone.0154635.s004.pdf]

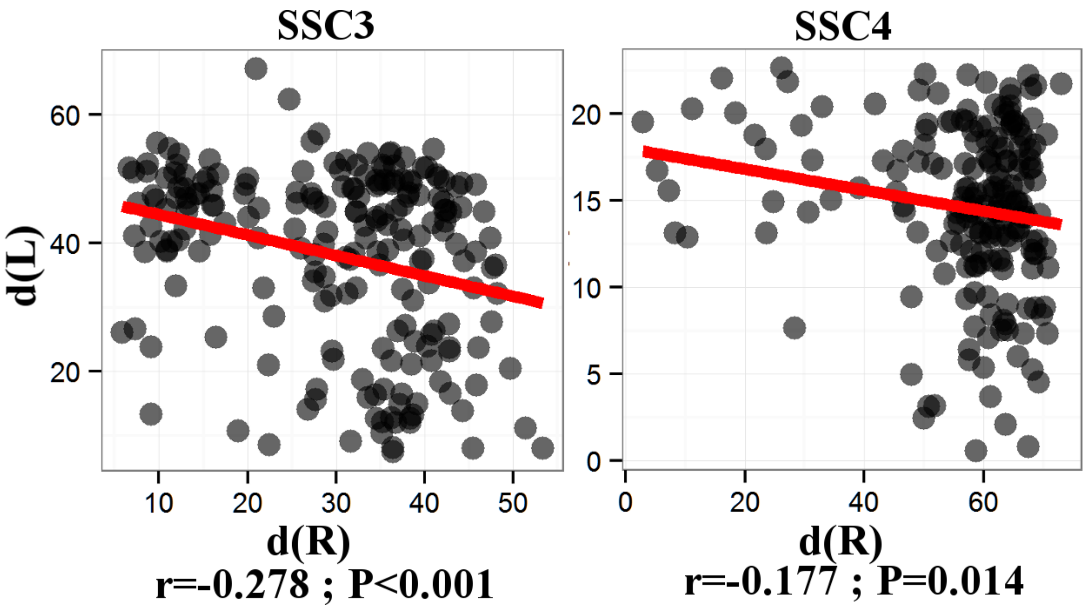

S4 Fig. Relationship between the two distances from the inferred "would-be breakpoint" to the nearest CO on the left [d(L)] and right [d(R)] sides of chromosomes that have at least one CO on each side for control boars. For the two chromosomes, results of correlation analyses are indicated. The distances were expressed as percentage of the SC length.
