# Supplementary material for: Meiotic Recombination Analyses in Pigs Carrying Different Balanced Structural Chromosomal Rearrangements
Source: PLoS One. 2016 Apr 28;11(4):e0154635. doi: 10.1371/journal.pone.0154635 (PMC4849707; doi:10.1371/journal.pone.0154635)
Supplement: S1 Table — (PDF) [file pone.0154635.s005.pdf]

S1 Table. Number of spermatocytes analyzed, mean MLH1 foci numbers and relative SC length per spermatocytes, and MLH1 distribution comparison.

		Mean number of MLH1 foci per spermatocyte (±SE)			Mean relative length[b] per spermatocyte (±SD)			MLH1 Distribution comparison[c]		
**Individuals**	**No. of cells[a]**	**SSC2**	**SSC8**	**SSC9**	**SSC2**	**SSC8**	**SSC9**	**SSC2**	**SSC8**	**SSC9**
Controls	264	1.99 (0.03)	1.86 (0.02)	1.90 (0.03)	6.63 (0.74)	5.29 (0.59)	5.14 (0.56)			
T34he	63	1.97 (0.09)	1.73 (0.06)	1.76 (0.07)	6.75 (1.02)	5.31 (0.55)	5.03 (0.65)	0.523	0.244	0.638

[a] number of spermatocytes analyzed by FISH (p and q arms identified for SSC2, SSC8 and SSC9).
[b] Percent of total autosomal SC length.
[c] p values from Kolmogorov-Smirnov tests (comparison between T34he and controls)
* P<0.01; ** P<0.001 compared to controls.
